# Is maternal diabetes during pregnancy associated with neurodevelopmental, cognitive and behavioural outcomes in children? Insights from individual participant data meta-analysis in ten birth cohorts

**DOI:** 10.1186/s12887-024-05365-y

**Published:** 2025-01-30

**Authors:** Rachelle A. Pretorius, Demetris Avraam, Mònica Guxens, Jordi Julvez, Jennifer R. Harris, Johanna Thorbjornsrud Nader, Tim Cadman, Ahmed Elhakeem, Katrine Strandberg-Larsen, Hanan El Marroun, Serena Defina, Tiffany C. Yang, Rosie McEachan, John Wright, Jesús Ibarluzea, Loreto Santa-Marina, Juana Mari Delgado, Marisa Rebagliato, Marie-Aline Charles, Chloe Vainqueur, Silvia Maritano, Daniela Zugna, Wen Lun Yuan, Barbara Heude, Rae-Chi Huang

**Affiliations:** 1 Nutrition & Health Innovation Research Institute, Edith Cowan University, Perth, WA Australia; 2 Medical School, The University of Western Australia, Crawly, Perth, WA Australia; 3 School of Medical, Molecular and Forensic Sciences, College of Environmental and Life Sciences, Murdoch University, Murdoch, Perth, WA Australia; 4 Department of Public Health, Policy and Systems, University of Liverpool, Liverpool, UK; 5 Section of Epidemiology, Department of Public Health, University of Copenhagen, Copenhagen, Denmark; 6 ISGlobal in Barcelona, Barcelona, Catalonia Spain; 7 Universitat Pompeu Fabra, Barcelona, Catalonia Spain; 8 Department of Child and Adolescent Psychiatry/Psychology, Erasmus MC, University Medical Centre, Rotterdam, The Netherlands; 9 Clinical and Epidemiological Neuroscience Group (NeuroÈpia), Institut d’Investigació Sanitària Pere Virgili (IISPV), 43204 Reus (Tarragona), Catalonia, Barcelona, Spain; 10 Centre for Fertility and Health, Norwegian Institute of Public Health, Oslo, Norway; 11 Department of Genetics and Bioinformatics, Division of Health Data and Digitalisation, Norwegian Institute of Public Health, Oslo, Norway; 12 UMCG Genetics Department, Genetics Department, University Medical Centre Groningen, GCC - Genomic Coordination Centre), Groningen, The Netherlands; 13 Population Health Science, Bristol Medical School, University of Bristol, Bristol, UK; 14 Department of Psychology, Education and Child Studies, Erasmus School of Social and Behavioural Sciences, Erasmus University Rotterdam, Rotterdam, The Netherlands; 15 The Generation R Study Group, Erasmus MC, University Medical Center Rotterdam, Rotterdam, The Netherlands; 16 Bradford Institute for Health Research, Bradford Teaching Hospitals NHS Foundation Trust, Bradford, UK; 17 Biogipuzkoa Health Research Institute, Environmental Epidemiology and Child Development San Sebastian, Madrid, Spain; 18 Spanish Consortium for Research on Epidemiology and Public Health (CIBERESP), Instituto de Salud Carlos III, Madrid, Spain; 19 Biodonostia Health Research Institute, Environmental Epidemiology and Child Development Group, San Sebastian, Madrid, Spain; 20 Department of Medicine, Faculty of Health Sciences, Universitat Jaume I, Avenida de Vicent Sos Baynat s/n, Castellón de la Plana, 12071 Spain; 21 Epidemiology and Environmental Health Joint Research Unit, Foundation for the Promotion of Health and Biomedical Research in the Valencian Region, FISABIO-Public Health, FISABIO–Universitat Jaume I–Universitat de València, Valencia, Spain; 22 Epidemiology and Environmental Health Joint Research Unit, FISABIO–Universitat Jaume I–Universitat de València, Valencia, Spain; 23 Université Paris Cité and Université Sorbonne Paris Nord, INRAE, Centre for Research in Epidemiology and StatisticS (CRESS), Inserm, Paris, F-75004 France; 24 Joint unit Elfe, Ined, Inserm, Aubervilliers, 93322 France; 25 Cancer Epidemiology Unit, Medical Science Department, University of Turin and CPO Piemonte, Via Santena 7, Turin, 10126 Italy; 26 University School for Advanced Studies IUSS, Palazzo del Broletto, Piazza della Vittoria, Pavia, PV 27100 Italy; 27 The Kids Research Institute Australia, The University of Western Australia, WA Perth, Australia

**Keywords:** Gestational diabetes mellitus, Attention deficient hyperactive disorder, Autism spectrum disorder, Neurodevelopmental, Cognitive, Behavioural, Externalising problems

## Abstract

**Background:**

Growing evidence shows that dysregulated metabolic intrauterine environments can affect offspring’s neurodevelopment and behaviour. However, the results of individual cohort studies have been inconsistent. We aimed to investigate the association between maternal diabetes before pregnancy and gestational diabetes mellitus (GDM) with neurodevelopmental, cognitive and behavioural outcomes in children.

**Methods:**

Harmonised data from > 200 000 mother-child pairs across ten birth cohorts in Europe and Australia were available. Mother-child pairs were included for analysis to determine whether GDM was recorded (yes or no) and whether at least one neurodevelopmental, cognitive and behavioural outcome was available in children aged 3 to 13 years. Confounder-adjusted regression models were used to estimate associations between maternal diabetes and child outcomes using two-stage individual participant data (IPD) meta-analysis. Model 1 included a crude estimate. The full adjustment model (model 2) included adjustment for child sex, maternal age, pre-pregnancy BMI, pregnancy weight gain, maternal smoking during pregnancy, plurality, parity and maternal education.

**Results:**

Children (aged 7–10 years) born to mothers with GDM had higher attention-deficient hyperactive disorder (ADHD) symptoms compared to non-exposed controls (model 2, regression coefficient (β) 3.67 (95% CI 1.13, 6.20), *P* = 0.001). Moreover, children (aged 4–6 years) born to mothers with GDM exhibited more externalising problems than those born to mothers without GDM (model 2, β 2.77 (95% CI 0.52, 5.02), *P* = 0.01). A pre-existing maternal history of type 1 and type 2 diabetes mellitus was associated with ADHD symptoms at 4–6 years (model 1, β 8.82 (95% CI 2.21, 15.45, *P* = 0.009) and β 7.90 (95% CI 0.82, 14.98, *P* = 0.02), respectively). The association was no longer apparent in further adjustments.

**Conclusions:**

This study found that children between 4 - 6 and 7–10 years of age born to mothers with GDM have a greater likelihood of developing externalising problems and ADHD symptoms, respectively. Externalising problems often co-exist with ADHD symptoms and precede formal ADHD diagnosis. Overall, this large-scale multi-cohort study suggested that a dysregulated metabolic environment during pregnancy may contribute to ADHD symptoms and externalising problems in young children.

**Supplementary Information:**

The online version contains supplementary material available at 10.1186/s12887-024-05365-y.

## Introduction

 Between 10% and 20% of children are affected annually by mental health, cognitive and behavioural disorders, with similar rates across different racial and ethnic groups after controlling for income, resident status, education, and neighbourhood support [1, 2]. This can manifest as internalising or externalising problems, delayed non-verbal intelligence, language, and gross and fine motor development in childhood. Specific neurodevelopmental disorders such as autism spectrum disorder (ASD) and attention deficit hyperactivity disorder (ADHD) are prevalent worldwide, affecting approximately 2.9% and 8.5% of children, respectively [3, 4]. ADHD is characterised by symptoms such as inattention, impulsivity, and hyperactivity and often co-occurs with ASD, which is characterised by a lack of social interaction as well as restrictive, repetitive patterns of behaviour, interest or activities [3, 5].

It is becoming increasingly evident that the *in-utero* metabolic milieu may impact brain development in offspring [6–10]. Exposure to diabetes before or during pregnancy, characterised by elevated blood glucose levels and placental inflammation responses, may impact early fetal brain development and result in delayed brain maturation [9, 11–16]. Type 1 and Type 2 diabetes mellitus (T1DM and T2DM) affects 529 million women worldwide [17], whereas diabetes during pregnancy (gestational diabetes mellitus (GDM)) affects approximately one in six women (17.9%) [18–20].

Xiang and colleagues (2023) studied the relation between maternal diabetes in pregnancy and neurodevelopmental disorders (ASD [12] and ADHD [21]) in offspring using health record data (*n* > 300 000) of children born at Kaiser Permanente Southern California (KPSC) from 1995 to 2009 with follow-up until 2012. No overall association was found between GDM and either ASD [21] or ADHD [34]. Timing and dose of exposure to GDM were explored. Timing appeared to play a role in the association with ASD but not with ADHD. An early GDM diagnosis (before 26 weeks gestation) was associated with an increased risk of ASD diagnosis [12, 21]. By contrast, no association was found between the timing of GDM exposure and ADHD [21]. Evidence for a dose response was also suggested. A higher maternal haemoglobin A_1c_ (HbA_1c_) level in early pregnancy was associated with an increased risk of ASD in the offspring, suggesting that glycaemic control in early pregnancy may be an important window for ASD risk in offspring [22]. In addition, a significant association was detected between mothers with GDM taking antidiabetic medication and ADHD in their children. No such association was present in the mothers with GDM not receiving medication [21].

Previous studies have also examined the relationship between maternal diabetes and cognitive and behavioural problems, such as motor development, intelligence, and internalising and externalising behaviours, with varying results [23–25]. In a meta-analysis by Arabiat et al. (2021) [23], it was found that children born to mothers with diabetes (during pregnancy and pre-existing) scored lower on tests of gross motor function compared with children born to mothers without diabetes. This study reported the weighted mean difference as − 0.75 (95% CI: − 1.29, − 0.21), with a *p*-value of 0.007 and an I^2^ of 24%. It’s important to note that the study did not separately analyse the association of T1DM, T2DM, and GDM with gross motor function. More recently, Faleschini et al. (2023) [37] studied 548 mother-child pairs from a prospective pre-birth Gen3G cohort in Canada and measured maternal glycaemic markers during pregnancy using an oral glucose tolerance test (OGTT). The authors report that exposure to GDM was associated with higher externalising scores at 3 and 5 years [β = 1.12, 95% CI: 0.14, 2.10] after adjustment for child sex, maternal body mass index and family history of diabetes [25]. The authors suggested an association between exposure to maternal GDM during pregnancy and greater levels of externalising behaviours in young children (3–5 years).

Given the variability in findings, longer follow-ups across the childhood period within large numbers are needed to evaluate if the associations persist or translate into other related outcomes. Well-designed international meta-analyses are required. Data linkage using healthcare data has used this approach, finding a small to moderate association between maternal diabetes mellitus and ADHD [26]. Our approach is to use two-stage individual participant data (IPD) meta-analysis applied on harmonised data, which provides reliable regression estimates by reducing between-study heterogeneity and allowing consistent adjustment for confounding factors [27]. Therefore, we aimed to use data from more than 200,000 mother-child dyads from ten different cohorts participating in the European Union Child Cohort Network (EUCCN) with rigorous harmonisation of GDM and mental health outcomes to investigate the association between diabetes before and during pregnancy and its potential impact on neurodevelopmental, cognitive, and behavioural outcomes in children between 3 and 13 years of age.

## Method

### Cohort studies and harmonisation of core variables

The study was part of the European Union-funded Horizon 2020 Project LifeCycle, with harmonised data from the EUCCN, an international collaboration between Australian and European birth cohort studies [28, 29]. The trial registration number for the project is ECCNLC202161 and the work was supported by funding from the Horizon 2020 LifeCycle (733206). The LifeCycle project has developed a protocol to generate with this aised variables across collected variables. Details of how variables were harmonised for LifeCycle are provided in a publicly available online catalogue (https://euchildcohortnetwork.eu/research-tools/) and elsewhere [28]. Jaddoe et al. 2020 [29] fully describe the work to achieve a harmonised set of FAIR (findable, accessible, interoperable, and reusable) data resources known as the EU Child Cohort Network (EUCCN).

Pregnancy and birth cohort studies from the EUCCN were eligible to participate if they had data on maternal GDM diagnosis (Yes or No) collected during pregnancy continuing beyond 24–28 weeks of gestation and data on at least one child’s neurodevelopmental, cognitive or behavioural outcome. Ten cohorts were eligible to participate in the study, and all agreed to participate in this analysis. These were ALSPAC (Avon Longitudinal Study of Parents and Children, United Kingdom, 1991–1992) [30, 31], BiB (Born in Bradford, United Kingdom, 2007–2011) [32], DNBC (Danish National Birth Cohort, Denmark, 1996–2003) [33], EDEN (study on the pre- & early postnatal determinants of child health & development, France, 2003–2006) [34], ELFE (The French National cohort of children, France, 2011–2016) [35], GenR (The Generation R Study, Rotterdam, the Netherlands, 2002–2006) [36], INMA (Environment and Childhood Project, Spain, 1997–2008) [37], MoBa (The Norwegian Mother, Father and Child Cohort Study, Norway, 1999–2008) [38], NINFEA (Nascita e INFanzia: gli Effetti dell’Ambiente, Italy, 2005–2016) [39] and Raine (The Raine Study, Australia, 1989–1991) [40]. All studies had ethical approval and obtained parental or participant written informed consent (Supplementary text 1).

### Exposures: maternal diabetes before and during pregnancy

Our primary exposure measure was a binary variable indicating the presence or absence of evidence for GDM. A secondary analysis was also performed using data about T1DM and T2DM before pregnancy. GDM, T1DM and T2DM were extracted from medical records, blood samples, OGTT, or maternal self-reporting results in questionnaires. Although, there is variability in ascertainment methods, each cohort harmonised their data according to the consortium’s protocol into a common data model format [28]. A binary variable (Yes or No) indicating the presence or absence of evidence for GDM was harmonised for each cohort based on extraction from clinical records or maternal self-report (Supplemental Table 1).

### Outcomes: child neurodevelopmental, cognitive and behavioural outcomes

We analysed data for seven neurodevelopmental and cognitive behavioural outcomes: ADHD symptoms, ASD symptoms, gross motor function, fine motor development, non-verbal intelligence, internalising, and externalising behaviours. These were core variables rigorously harmonised by strict protocols. A full list of the neurodevelopmental and cognitive behavioural outcomes harmonised in the EUCCN can be found in Work Package 6 of LifeCycle (https://euchildcohortnetwork.eu/research-tools/).

This study grouped outcomes in four age ranges: 3 years, 4–6 years, 7–10 years, and 11–13 years, broadly representing the stages of toddler preschool, school entry, late childhood and early adolescence. Age groups were selected to maximize the use of the available data from the various follow-up points while simultaneously considering childhood developmental stages and mirror prior analyses on these data [41]. 

A recent publication by Nader et al. 2023 [42] provides a detailed overview of the major mental health measures available in the LifeCycle project. ADHD percentile scores were measured using the Child Behaviour Checklist (CBCL) [43], Revised Conners’ Parent Rating Scale (CPRS-R) [44], Diagnostic Interview Schedule for Children (DISC-IV/DSM) [45], Teacher’s Report Form (TRF) [46] and Strengths and Weaknesses of ADHD Symptoms and Normal Behaviour (SWAN) [47]. ASD was measured by several instruments and medical records including the Alarm Distress Baby Scale (ADBB) [48], Autism Quotient Questionnaire (AQ) [49], Childhood Autism Spectrum Test (CAST) [50], Social Responsiveness Scale (SRS) [51], the Early Screening of Autistic Traits Questionnaire (ESAT) [52], the Non-Verbal Communication Checklist (NVCC) [53], and the Social Communication Questionnaire (SCQ) [54].

Gross and fine motor function were assessed using various instruments across the cohorts. They included the Ages and Stages Questionnaire (ASQ) [55], Peg Moving Task (PMT) [56], Brunet-Lezine psychometric scale (BDIST) [57], Bayley Scale of Infant Development (BSID) [58], Child Development Inventory (CDI) [59], Developmental Coordination Disorder Questionnaire (DCDQ) [60], Denver Development Screening Test (DDST) [61], Movement Assessment Battery for Children (M-ABC) [62], Children’s developmental progress from birth to five years (STYCAR) [63], McCarthy Scales of Children’s Abilities (MSCA) [64].

Internalising and externalising problems were measured using Child Behaviour Checklist (CBCL) [65], Strengths and Difficulties Questionnaire (SDQ) [66]. Measurement tools used to measure nonverbal intelligence included Ages and Stages questionnaires (ASQ) [55, 67], British Ability Scale (BAS) [68], Snijders-Oomen Non-verbal Intelligence Test (SON-R) [69], Bayley Scale of Infant Development (BSID) [58], Culture Fair Intelligence Test (CFIT) [70], Cartell Infant Intelligence Scale (CIIS) [71], McCarthy Scales of Children’s Abilities (MSCA) [64], Snijders-Oomen Non-Verbal Intelligence Test (SON-R) [69], Wechsler Intelligence Scale for Children (WISC) [72].

Table 1 shows which cohorts have data on neurodevelopmental, cognitive, and behavioural outcomes (measured as percentile scores) for each age group and the number of mother-child pairs included in each case.

**Table 1:** Availability of neurodevelopmental, cognitive and behavioural data per age group in each participating cohort

	Age group (in years)	Number of mother-child pairs	Percentile scores Median (IQR)	ALSPAC	BiB	DNBC	EDEN	ELFE	GenR	INMA	MoBa	NINFEA	Raine
**ADHD Symptoms**	3												
	4–6	72 563	47.37 (23.52, 67.78)	✓			✓	✓		✓	✓	✓	✓
	7–10	113 707	45.24 (22.49, 73.50)	✓		✓			v		✓		✓
	11–13	9861	42.93 (16.23, 67.30)	✓		✓						✓	✓
**ASD Symptoms**	3	57 939	24.34 (10.00, 60.67)								✓		
	4–6	17 893	48.79 (24.75, 74.79)						✓	✓	✓		
	7–10	42 660	46.00 (15.00, 71.00)								✓		
	11–13												
**Gross** **motor development**	3	69 714	7.58 (3.10, 12.20)				✓	✓			✓		
	4–6	13 306	20.27 (6.18, 33.22)				✓	✓		✓		✓	
	7–10	42 660	44.0 (25.00, 71.00)			✓							
	11–13												
**Fine** **motor development**	3	67 955	47.33 (4.80, 71.95)				✓	✓		✓	✓		
	4–6	10 567	38.8 (11.62, 42.67)				✓	✓		✓			✓
	7–10	58 790	45.46 (24.38, 71.28)				✓			✓			✓
	11–13												
**Non-verbal intelligence**	3												
	4–6	4986	53.34 (25.28, 74.63)	✓					✓	✓			✓
	7–10	9098	51.21 (27.22, 76.76)	✓						✓			✓
	11–13												
**Externalising problems**	3	67 083	47.43 (25.27, 75.00)	✓			✓	✓	✓		✓		
	4–6	61 115	41.09 (24.47, 72.34)	✓	✓		✓	✓	✓		✓		✓
	7–10	64 405	43.07 (14.39, 70.13)			✓	✓		✓	✓			
	11–13	57 175	48.82 (19.00, 72.42)	✓		✓							
**Internalising problems**	3	66 339	42.54 (18.63, 63.25)	✓			✓	✓	✓		✓		
	4–6	70 354	46.81 (21.16, 73.39)	✓	✓		✓	✓	✓		✓		✓
	7–10	56 938	47.00 (1.00, 64.00)			✓			✓				
	11–13	9908	45.73 (12.63, 72.84)	✓		✓						✓	✓

*Abbreviations: ALSPAC *Avon Longitudinal Study of Parents and Children, *BiB *Born in Bradford, *DNBC *Danish National Birth Cohort, *EDEN *study on the pre- & early postnatal determinants of child health & development, *ELFE *The French National cohort of children, *GenR *The Generation R Study, *INMA *Environment and Childhood Project, *MoBa *The Norwegian Mother, Father and Child Cohort Study, *NINFEA *Nascita e INFanzia: gli Effetti dell’Ambiente, *Raine *The Raine Study, *IQR *Inter Quartile range

Percentile scores were calculated for each cohort and data collection wave separately to compare the outcomes on the same scale (rather than the original scale of the different instruments) [42, 73]. A percentile score indicates a child’s relative position within his/her cohort and age group [42]. The harmonisation process under the LifeCycle project allows meta-analysis of data initially collected using different scales or instruments [28]. 

### Confounders

Potential confounders were identified based on the literature [74–76] and these measures were harmonised across cohorts. Two models of estimates were used. Model 1 was crude and model 2 was adjusted for the following confounders: maternal gestational weight, pre-pregnancy body mass index (BMI), maternal smoking during pregnancy, parity (number of times giving birth), plurality, maternal education and household income. Information on the confounders, including child sex and maternal smoking during pregnancy, was obtained from hospital records and/or questionnaires. Maternal pre-pregnancy BMI was determined by weight and height at the first visit. Maternal education variable was harmonised across cohorts based on the International Standard Classification of Education 97 (ISCED-97) and consisted of three categories: Low (No education to lower secondary; ISCED‐97 categories 0‐2), Medium (Upper and post‐secondary; ISCED‐97 categories 3‐4), High (Degree and above; ISCED‐97 categories 5‐6) [76, 77]. For the sensitivity adjustment (model 3), the EU statistics on income and living conditions (EU-SILC) were added to collect timely and comparable cross-sectional and longitudinal data on income, poverty, social exclusion, and living conditions [76]. The Raine Study was excluded from analysis model 3 due to the absence of EU-SILC income data.

### Statistical analysis

The two-stage IPD meta-analysis examined the relationship between GDM and neurodevelopmental, cognitive and behavioural outcomes in children aged 3, 4–6, 7–10, and 11–13 years. A regression model is fitted on the data of each cohort separately and the cohort-specific estimates are then combined with a random-effects meta-analysis. We used the rma function from the metafor R package (version 4.6-0) with the Restricted Estimate Maximum Likelihood method for the random-effects meta-analysis. With this method, the combined estimate is given as the weighted average of the cohort-specific estimates where the weights are defined as the inverse of the variance of the estimates.

We employed linear regression models since percentile scores were used for the outcome variables. Each regression was performed on the complete cases for each set of variables (exposure, outcome, and confounders). Therefore, data about children with at least one missing value for any variable included in a model were excluded from the analysis. Regression models were fitted separately for each cohort, and regression coefficients (β) and standard errors (SE) were combined using random effects meta-analysis with the restricted maximum likelihood estimator method [78]. Between-cohort heterogeneity was evaluated by *I*^*2*^ and Q statistics. In the secondary analysis, we examined the associations of maternal T1DM and T2DM diabetes before pregnancy with percentile scores of ADHD symptoms.

All analyses were run on DataSHIELD (R packages dsBaseClient v6.1.0 & dsHelper v1.1.0), a platform allowing privacy-preserving co-analysis of data from multiple cohorts without the need to share or transfer the individual-level data [79, 80].

## Results

Table 2 summarises the maternal characteristics of each cohort. Mean maternal age ranged between 27 and 33 years. Mean maternal BMI was 23.2 kg/m^2^. EDEN, ELFE, and MoBa had the highest proportion of mothers with higher education, at 53.5%, 56.6%, and 64.2%, respectively. Raine study, ALSPAC and BiB had a lower proportion of mothers with higher education, at 19.4%, 13.2% and 27.6% respectively. A high rate of vaginal birth was observed among the cohorts, the exceptions being MoBa, BiB and The Raine study having rates below 70%.

In total, 266 970 pregnant women across the ten cohorts had information on the presence or absence of GDM. The prevalence of GDM differed between cohorts, ranging from 0.65% (NINFEA) to 8.01% (BiB) (Table 2). The prevalence of T1DM ranged from 0.003% (NINFEA) to 0.24% (ALSPAC), and of T2DM ranged from 0.00% (NINFEA) to 0.25% (ELFE). A total of 132,249 pregnant mothers across four cohorts (ALSPAC, BiB, DNBC, and ELFE) had data available on maternal T1DM and T2DM before pregnancy. Overall, the distribution of birth weight and head circumference were similar across the cohorts, with a combined median of 3371 g (interquartile range (IQR): 3038, 3855) for birth weight and 33.88 cm (IQR: 34.86, 35.88) for head circumference at birth.

**Table 2:** Maternal-related characteristics of each participating cohort

	Total	ALSPAC	BiB	DNBC	EDEN	ELFE	GenR	INMA	MoBa	NINFEA	Raine
**Maternal age (years)**	29.80 (26.86–33.03)	28.00 (25.00–32.00)	27.00 (23.00–31.00)	30.00 (27.00–33.00)	29.00 (26.00–33.00)	30.00 (27.00–34.00)	31.00 (27.00–34.00)	32.00 (29.00–34.00)	30.00 (27.00–33.00)	33.00 (30.00–36.00)	28.00 (23.00–32.00)
**Parity (0)**	133,302/272,464 (48.9%)	5869/17,697 (33.2%)	5170/13,029 (39.7%)	46,250/96,825 (47.8%)	848/1903 (44.5%)	8249/17,957 (45.9%)	5239/9522 (39.7%)	1168/2118 (55.1%)	48,649/103,419 (47.0%)	5184/7189 (72.1%)	1342/2804 (46.8%)
**Vaginal birth**	185,218/237,810 (77.9%)	10,518/11,962 (87.9%)	7475/10,959 (85.0%)	71 954/96,181 (74.8%)	1045/1733 (81.1%)	12,172/17,735 (68.6%)	6224/8455 (73.6%)	1216/1948 (87.9%)	69,200/81,377 (65.4%)	4354/6577 (57.0%)	2006/2831 (69.9%)
**Education after high school**	133,958/250,899 (53.4%)	1609/12,483 (13.2%)	2920/10,563 (27.6%)	42,518/84,136 (50.5%)	1021/1910 (53.5%)	10,319/18,218 (56.6%)	3713/8661 (42.9%)	710/2176 (32.6%)	66,081/102,919 (64.2%)	4579/7176 (63.8%)	515/2657 (19.4%)
**EU-SILC**^**^^**^	7.8 (7.7–7.9)	7.1 (6.9–7.2)	6.9 (6.7–7.1)	7.9 (7.3–8.1)	7.4 (7.1–7.6)	7.4 (7.2–7.7)	8.1 (7.9–8.1)	7.1 (6.8–7.3)	8.1 (7.9–8.1)	7.4 (7.3–7.6)	NA
**Weight (kg)**^a^	64.0 (57.6–72.5)	58.42 (52.49–58.42)	65.0 (56.0–76.0)	65.0 (58.0–73.0)	60.0 (54.0–68.0)	61.0 (55.0–70.0)	64.0 (53.0–72.0)	60.0 (54.2–67.9)	65.0 (59.0–74.0)	59.0 (54.0–67.0)	57.0 (52.0–65.0)
**BMI**,** kg/m**^**2a**^	23.2 (20.7, 24.1)	20.8 (19.7, 22.8)	26.4 (21.6, 27.5)	23.9 (20.3, 24.4)	22.5 (21.4, 24.2)	23.8 (20.2, 24.2)	24.4 (19.0, 24.1)	23.7 (20.4, 24.3)	23.0 (21.9, 25.0)	21.7 (21.1, 23.2)	21.2 (20.3, 23.0)
**Pregnancy** **weight gain (kg)**	14.7 (11.28–17.87)	16.4 (13.1–19.7)	NA	15.0 (12.0–18.0)	13.0 (10.0–16.0)	13.0 (10.0–16.0)	15.0 (11.0–18.0)	13.5 (10.5–16.6)	15.0 (11.0–18.4)	12.0 (10.0–15.0)	8.6 (6.5–11.0)
**DIABETES DURING PREGNANCY**											
**T1DM (yes)**^a^	392/132,246 (0.3%)	2911/11,941 (0.2%)	19/5729 (0.3%)	261/96,879 (0.03%)	NA	83/17,697 (0.5%)	NA	NA	NA	NA	NA
**T2DM (yes)**^a^	165/132,246 (0.1%)	20/11,941 (0.2%)	11/5729 (0.2%)	51/96,879 (0.1%)	NA	83/11,941 (0.5%)	NA	NA	NA	NA	NA
**GDM (yes)**	4555/266,970 (1.7%)	58/12,408 (7.0%)	1077/13,436 (8.0%)	868/96,822 (0.9%)	123/1904 (6.5%)	1213/17,305 (7.0%)	105/9192 (1.1%)	85/2010 (4.2%)	480/103,419 (0.1%)	491/7606 (0.7%)	55/2868 (1.9%)

Values are median (IQR) or n (percent)

*Abbreviations: ALSPAC *Avon Longitudinal Study of Parents and Children, *BiB *Born in Bradford, *DNBC *Danish National Birth Cohort, *EDEN *study on the pre- & early postnatal determinants of child health & development, *ELFE *The French National cohort of children, *GenR *The Generation R Study, *INMA *Environment and Childhood Project, *MoBa *The Norwegian Mother, Father and Child Cohort Study, *NINFEA *Nascita e INFanzia: gli Effetti dell'Ambiente and Raine, The Raine Study, *NA *Data not available for the cohort

^^^^ EU-SILC, comparable cross-sectional and longitudinal data on income, poverty, social exclusion, and living conditions

^a^characteristic before pregnancy

### Association of GDM with neurodevelopmental, cognitive and behavioural outcomes

Table 3 shows the adjusted regression estimates (β) of the association between GDM and neurodevelopment, cognitive and behavioural outcomes (measured as percentile scores) at different ages derived from the two-stage IPD meta-analysis.

### GDM and ADHD

**Fig. 1:**
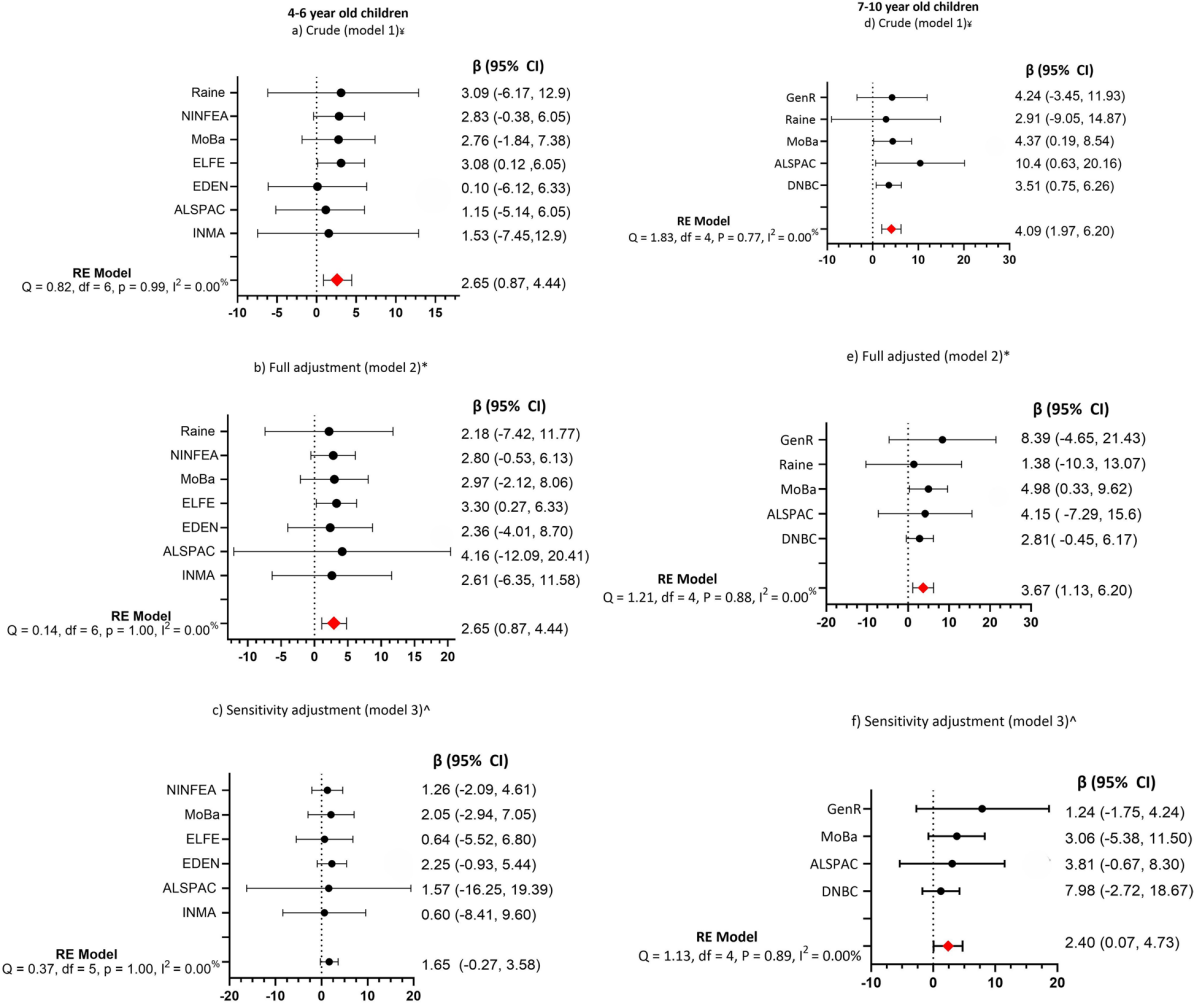
Association between GDM and offspring’s ADHD symptoms at 4–6 and 7–10 year of age. The forest plot shows the Regression Coefficient (β) and random effect (RE) for ADHD percentiles. Model 1 (**a**) and (**d**) include crude estimates, model 2 (**b**) and (**e**) full adjustment for child sex, maternal age, plurality and parity, BMI, pregnancy weight gain, and maternal smoking, and sensitivity adjustments (model 3 (**c**) and (**f**)) include full adjustment and EU-SILC. The Sensitivity adjustment excludes the Raine Study

 In the crude estimates (models 1), children aged 4–6 years born to mothers with GDM had significantly higher percentile scores of ADHD symptoms than those born to mothers without GDM (β =2.65 (95% CI:0.87, 4.44) *P* = 0.004). After full adjustment (model 2), the associations retain significance (β =2.96 (95% CI:1.10, 4.81) *P* = 0.001). The association was no longer apparent after the sensitivity analysis (model 3) (β =1.65 (95% CI: −0.27, 3.58) *P* = 0.09). Notably, significance remained unchanged in children aged 7–10 years in crude (model 1) and adjusted estimates (model 2 and 3), demonstrating that children born to mothers with GDM had significantly higher percentile scores of ADHD symptoms than children born to mothers without GDM (model 1: β =4.09 (95% CI: 01.97, 6.20) (*P* < 0.001), (β =3.67 (95% CI: 1.13, 6.20) *P* = 0.001)and model 3: β = 2.40 (95% CI: 0.07, 4.73) *P* = 0.04). The I^2^ was 0.00% for the three adjusted models at both age groups (4–6 and 7–10 years) (Fig. 1). There was no significant association between GDM and ADHD in children between 11 and 13 years of age.

### GDM and ASD

In the crude estimates (model 1) mothers with GDM tend to have children who exhibit more ASD symptoms at ages 4–6 and 7–10 compared to mothers without GDM (β = 6.09 (95% CI: 1.03, 11.15) *P* = 0.01) and (β = 4.42 (95% CI: 0.11, 8.73) *P* = 0.04), respectively. After full adjustment (model 2) and sensitivity analysis (model 3), the associations diminished in children 4–6 years.

### GDM and other neurodevelopmental, cognitive and behavioural symptoms

**Fig. 2:**
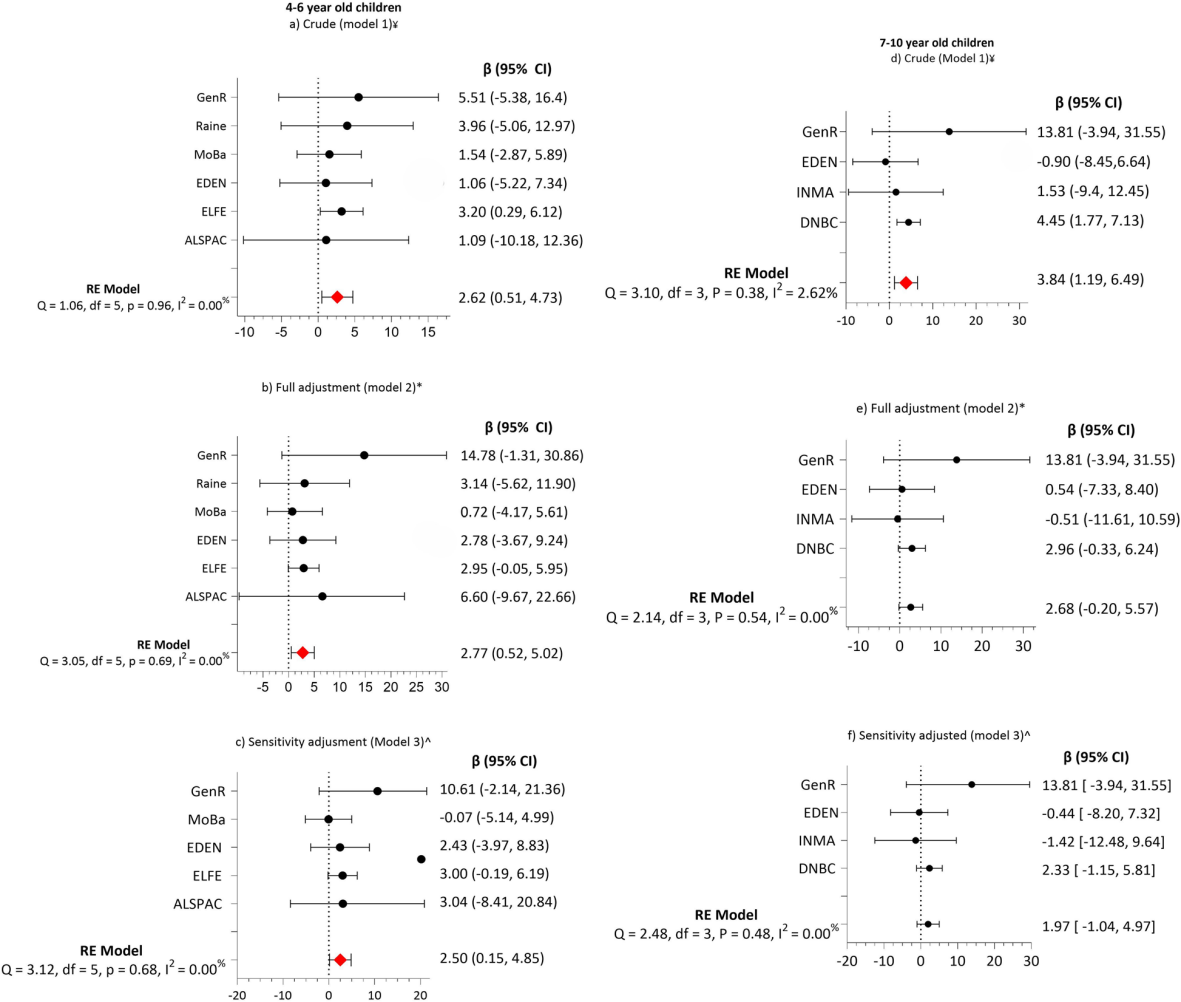
Association between GDM and offspring’s externalising problems at 4-6 and 7-10 year of age. The forest plot is showing Regression Coefficient (β) and random effect (RE) for externalising problems in children 4–6 and 7–10 years of age exposed to GDM versus children not exposed to GDM. Model 1 (**a**) and (**d**) include crude estimates, model 2 (**b**) and (**e**) full adjustment for child sex, maternal age, plurality and parity, BMI, pregnancy weight gain, maternal smoking and sensitivity adjustments (model 3 (**c**) and (**f**)) include full adjustment and EU-SILC. The Sensitivity adjustment excludes the Raine Study

GDM was not significantly associated with any changes in motor function (gross and fine) and nonverbal intelligence in children of any age in all three adjusted models (Table 3). However, children aged 4–6 years born to mothers with GDM consistently exhibited more externalising problems than those born to mothers without GDM in crude estimates (model 1) (β =2.62 (95% CI: 0.51, 4.73) *P* = 0.01), full adjustment (model 2) (β =2.77 (95% CI: 0.52, 5.02) *P* = 0.01) sensitivity analysis (model 3) (β =2.50 (95% CI: 0.15, 4.85) *P* = 0.03). Low heterogeneity was present among the cohorts for this outcome (range I^2^: 0.00–3.4%) (Fig. 2). Externalising problems were present among children aged 7–10 years born to mothers with GDM in the crude estimates (model 1) (β 3.84 (95% CI 1.19, 6.49) *P* = 0.005). However, the association was no longer apparent after full adjustment (model 2) and sensitivity adjustment (model 3). There was no association between externalising problems in children (11–13 years old) and GDM. Similarly, GDM was associated with more internalising problems among children 7–10 years in the crude estimates (model 1) (β =5.65 (95% CI: 2.81, 8.50) *P* < 0.001). However, like externalising problems, the association diminished after further adjustment (models 2 and 3) for children between 7 and 10 years. For 11–13-year-old children born to mothers with GDM also showed significantly more internalising problems compared to children born to mothers without GDM in the crude estimates (model 1)(β =5.65 (95% CI: 0.40, 11.10) *P* = 0.03). However, the association was no longer apparent after further adjustments (models 2 and 3) (Table 3).

**Table 3:** Combined estimates and 95% CI from the two-stage IPD meta-analysis of the effect of maternal GDM on child neurodevelopmental, cognitive and behavioural outcomes at different ages derived from crude estimates (model 1), full adjusted estimates (model 2) and sensitivity analysis (model 3)

	ADHD symptoms	ASD symptoms	Gross motor function	Fine motor function	Non-verbal intelligence	Externalising problems	Internalising problems
	β (95% CI) P value	β (95% CI) P value	β (95% CI) P value	β (95% CI) P value	β (95% CI) P value	β (95% CI) P value	β (95% CI) P value
**Year 3**							
Model 1^a^		−0.80 (−4.81, 3.20) 0.69	−1.63 (−5.31, 2.06) 0.38			1.83 (−1.09, 4.76) 0.22	6.29 (−3.00, 15.59) 0.18
Model 2^b^		−0.71 (−5.25, 3.82) 0.75	1.71 (−5.35, 1.92) 0.35			0.72 (−2.53, 3.97) 0.66	5.66 (−3.30, 14.63) 0.21
Model 3^c^		−1.42 (−6.14, 3.29) 0.55	−1.47 (−5.17, 2.22) 0.43			0.61 (−2.74, 3.95) 0.77	3.37 (−3.41, 10.16) 0.33
**Year 4–6**							
Model 1^a^	**2.65 (0.87**,** 4.44)**	**6.09 (1.03**,** 11.15) 0.01**	−0.29 (−4.12, 3.54) 0.88	−1.42 (−3.46, 0.62) 0.17	−2.47 (−13.22, 8.28) 0.65	**2.62 (0.51**,** 4.73) 0.01**	0.69 (−1.79, 3.17) 0.59
Model 2^b^	**2.96 (1.10**,** 4.81) 0.001**	4.74 (−0.99, 10.47) 0.10	0.06 (−3.21, 1.07) 0.88	−0.44 (−1.90, 1.02) 0.55	0.92 (−5.64, 7.49) 0.78	**2.77 (0.52**,** 5.02) 0.01**	1.26 (−1.05, 3.57) 0.28
Model 3^c^	1.65 (−0.27, 3.58) 0.09	4.68 (−0.89, 10.25) 0.10	0.17 (−3.00, 3.34) 0.91	−0.34 (−1.51, 0.82) 0.56	2.30 (−4.25, 8.84) 0.49	**2.50 (0.15**,** 4.85) 0.03**	1.06 (−1.44, 3.55) 0.40
**Year 7–10**							
Model 1^a^	**4.09 (1.97**,** 6.20) <0.001**	**4.42 (0.11**,** 8.73) 0.04**	−0.97 (−3.02, 1.07) 0.35	− 1.03 (−3.03, 0.97) 0.31	0.42 (−17.37, 18.20) 0.96	**3.84 (1.19**,** 6.49) 0.005**	**5.65 (2.81**,** 850) <0.001**
Model 2^b^	**3.67 (1.13**,** 6.20) 0.001**	2.97 (−1.85, 7.79) 0.22	−0.24 (−2.74, 2.26) 0.85	−0.11 (−2.55, 2.34) 0.93	2.46 (−3.19, 8.13) 0.39	2.68 (−0.20, 5.57) 0.06	2.43 (−1.07, 5.93) 0.17
Model 3^c^	**2.40 (0.07**,** 4.73) 0.04**	3.43 (−1.52, 8.28) 0.17	1.29 (−1.38, 3.97) 0.34	1.16 (−1.53, 3.86) 0.39	2.78 (−4.37, 9.93) 0.12	1.97 (−1.04, 4.97) 0.20	0.29 (−3.42, 4.01) 0.87
**Year 13 − 11**							
Model 1^a^	−4.88 (−13.44, 3.68) 0.26					4.99 (−5.13, 15.11) 0.33	**5.65 (0.40**,** 11.10) 0.03**
Model 2^b^	(−15.09, 3.51) 0.22					5.59 (−15.93, 27.10) 0.61	1.07 (−4.37, 6.51) 0.70
Model 3^c^	−4.67 (−11.02, 1.69) 0.15					5.15 (−15.43, 25.72) 0.62	0.33 (−5.23, 5.88) 0.90

^a^Crude estimates

^b^Fully adjusted for child sex, maternal age, plurality and parity, BMI, pregnancy weight gain, maternal smoking, maternal education

^c^Sensitivity analysis (Fully adjusted for child sex, maternal age, plurality and parity, BMI, pregnancy weight gain, maternal smoking, maternal education and EU-SILC income. Raine was excluded from the sensitivityanalysis

**Fig. 3:**
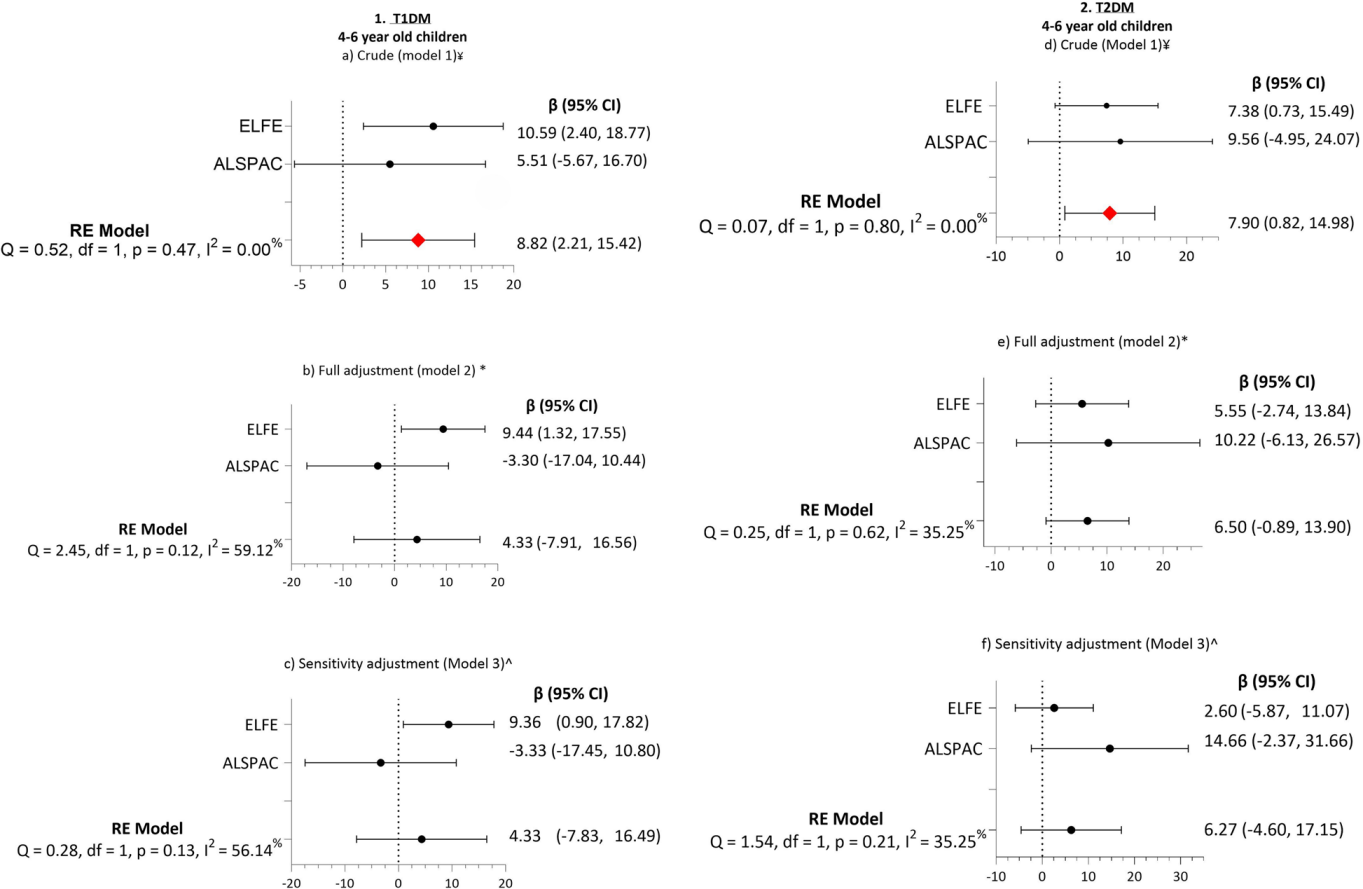
Association between T1DM and T2DM and offspring’s ADHD symptoms at 4–6 year of age. Forest plot showing Regression Coefficient (β) and random effect (RE) for ADHD percentile in children (4–6 years)) exposed to (1) T1DM and (2) T2DM versus those not exposed to T1DM and T2DM, respectively. Model 1 (**a**) and (**d**) include crude estimates, model 2 (**b**) and (**e**) full adjustment for child sex, maternal age, plurality and parity, BMI, pregnancy weight gain, maternal smoking and sensitivity adjustments (model 3 (**c**) and (**f**)) include full adjustment and EU-SILC. The Sensitivity adjustment excludes the Raine Study

 In a secondary analysis, the association between maternal diabetes before pregnancy (excluding GDM) and child ADHD symptoms was examined. In the crude estimates (model 1) children born to mothers with T1DM and T2DM had more significant ADHD symptoms than their counterparts at 4–6 years of age compared to the children born to mothers without diabetes before pregnancy (model 1) ((β =8.82 (95% CI: 2.21, 15.42) *P* = 0.009) and (β = 7.90 (95% CI: 0.82, 14.98) *P* = 0.02 )) (Table 4). The effects diminished after full adjustment (models 2) for both T1DM (β = 4.33 (95% CI −7.91, 16.56) *P* = 0.48) and T2DM (β = 6.50 (95% CI: −0.89, 13.90) *P* = 0.08) and after sensitivity analysis (model 3) (β = 4.33 (95% CI: −7.83, 16.49) *P* = 0.48) and T2DM (β = 6.27 (95% CI: −4.60, 17.15) *P* = 0.25). There was no association between GDM and ADHD in any adjusted models in children aged 7–10. In Model 2, the heterogeneity increased in both age groups compared to model 1 (Fig. 3).

**Table 4:** Combined estimates and 95% CI from the two-stage IPD meta-analysis of the effect of maternal T1DM and T2DM on child ADHD symptoms a 4–6 and 7–10 years derived from crude estimates (model 1), full adjusted estimates (model 2) and sensitivity analysis (model 3)

	T1DM	T2DM
	β (95% CI) P value	β (95% CI) P value
**Year 4–6**		
Model 1^a^	**8.82 (2.21, 15.45) 0.009**	**7.90 (0.82**,** 14.98) 0.02**
Model 2^b^	4.33 (−7.91, 16.56) 0.48	6.50 (−0.89, 13.90) 0.08
Model 3^c^	4.33 (−7.83, 16.49) 0.48	6.27 (−4.60, 17.15) 0.25
**Year 7–10**		
Model 1^a^	3.14 (−3.68, 9.96) 0.34	5.05 (−4.12, 14.22) 0.28
Model 2^b^	−0.28 (−5.45, 4.89) 0.91	5.85 (−7.82, 19.53) 0.40
Model 3^c^	0.12 (−5.40, 5.64) 0.96	8.87 (−2.74, 20.47) 0.13

^a^Crude estimates

^b^Fully adjusted for child sex, maternal age, plurality and parity, BMI, pregnancy weight gain, maternal smoking, maternal

^c^Sensitivity analysis (fully adjusted for child sex, maternal age, plurality and parity, BMI, pregnancy weight gain, maternal smoking,maternal education and EU-SILC income. Raine was excluded from the sensitivity analysis

## Discussion

In this study, a consistent finding in the crude and fully adjusted models was that children aged 7–10 years who were exposed to GDM had more ADHD symptoms than children born to mothers without GDM. We also found that children between the ages of 4–6 years born to mothers diagnosed with GDM exhibited higher externalising problems compared to those born to mothers without GDM after adjustments. The results suggest that GDM may be linked to ADHD symptoms in older children as well as externalising problems that often co-occur with ADHD in younger children. This is the first and the largest IPD meta-analysis using harmonised individual-level data that has investigated the association between maternal diabetes before and during pregnancy with neurodevelopmental, cognitive and behavioural outcomes in children from 3 years up to 13 years.

These results corroborate the American data from the Kaiser Permanente study [12, 21] which showed that a significant association was detected between some mothers with GDM and ADHD in their children, with the association being restricted to mothers with GDM taking antidiabetic medication. Our study has consolidated this finding and extended this more generally, such that the association persists among all women with GDM. This confirms the findings from another recent multinational meta-analysis [26], further confirming the robust association between GDM and ADHD.

Cognitive, emotional, and behavioural difficulties first emerge in early childhood, laying the foundation for continued or increasing problems during middle and late childhood [81]. Still, the mechanisms underlying these longitudinal associations or co-development between neurodevelopment, cognitive and behavioural domains from early childhood to early adulthood remain poorly elucidated [82]. Our finding that GDM is associated with externalising outcomes in young children (4–6 years) is consistent with the results of a previous study by Faleschini et al. [25], who found an association between GDM and externalising behaviours in young children at age 3 and 5 years. Interestingly, while we tested the associations at different age ranges, our significant finding was at the same approximate age as the Canadian study, around 3–6 years, suggesting that this is a sensitive age range for detecting this childhood symptomatology. ADHD does not have biological markers for diagnosis, making ADHD a disorder that is difficult to detect before symptoms manifest [83, 84]. However, our findings suggest that these externalising behaviours co-develop and extend into other domains, such as ADHD symptoms. We postulate that children may exhibit more externalising problems at younger ages and that as they mature, symptoms or behaviour related to ADHD may become more apparent.

Of note, our findings were attenuated with adjustment, particularly with a measure of the family’s socio-economic status. Nomura et al. (2012) [85] reported that children exposed to both GDM, and low socioeconomic status had a 14-fold increase in the risk for ADHD compared to those exposed GDM or low socioeconomic status (SES) alone. More recently, Cadman et al. (2024) [86]. Showed in their longitudinal study that children born into more disadvantaged socioeconomic status had more behavioural and cognitive problems. While women with low socioeconomic status are more likely to have more severe hyperglycaemia, which may affect the neurodevelopment of their children, household SES may also reflect poorer diet, greater maternal and child obesity, poorer health literacy, lesser early educational opportunities or other unmeasured factors. Recent studies show that siblings with discordant exposure to GDM in pregnancy had a similar risk of ADHD [26]. Indeed, such factors and shared genetics or familial factors between mother and offspring may partially or fully confound the association.

Increased inflammation, oxidative stress, hypoxia, and hyperinsulinemia during pregnancy may influence certain pathways in a child’s brain programming *in-utero* and contribute to neurodevelopmental, cognitive and behavioural outcomes later in life [21, 87–89]. Several studies suggest that maternal obesity, chronic inflammation, and maternal diabetes have a joint impact on the development of ASD and ADHD in children, which is greater than the impact of either condition alone [11, 90–93]. Additionally, it has been observed that the extent of diabetes (T1DM vs. T2DM vs. GDM requiring antidiabetic medication due to severe hyperglycaemia) during pregnancy has a more significant impact on the risk of ADHD symptoms. On the other hand, the timing of maternal diabetes does not influence ADHD symptoms [21].

This is a large two-staged meta-analysis using IPD to examine associations between maternal diabetes before and during pregnancy and various determinants of neurodevelopmental and cognitive and behavioural outcomes in children at various age groups. Our study has the following strengths. First, two-stage meta-analysis of IPD has advantages over the meta-analysis of published aggregate data as it avoids potential publication bias and reduces between-study heterogeneity by using harmonised data [94]. Second, the harmonised data on GDM diagnosis, neurodevelopmental, and cognitive and behavioural outcomes ensured comparability across cohorts. Third, the federated analysis using the DataSHIELD ensures that all analysis is performed identically, eliminating the need for individual researchers within each cohort to run analysis scripts. Fourth, combining data from 10 cohorts leads to larger numbers, providing the opportunity to increase statistical powder and obtain more precise estimates than any single cohort [86, 95]. Finally, replicating findings across diverse populations with varying cultural and socio-economic backgrounds enhances our confidence that the findings are applicable to a broader demographic, reinforcing the generalizability [28].

Using data from the EUCCN provided opportunities but also challenges. Our study has certain limitations. First, while GDM data were rigorously harmonised, the method of ascertainment varied (as reported in Supplementary Table 1). We particularly acknowledge the risk of bias when using parent-reported measures of cognitive and behavioural outcomes, which may have the potential for overestimation or proxy-reporting bias. Nevertheless, research suggests that parent-reported cognitive abilities can effectively assess cognitive and behavioural function when formal assessments are unavailable [96]. Attrition is a limitation in all studies that collect data over the years, and some cohorts may lack the data required to harmonise variables. However, in our study, we employed a complete case analysis to ensure that the results were not biased. Finally, confounding due to potentially unmeasured factors (such as factors related to SES and maternal ADHD) could not be ruled out.

To progress further in the field of child psychiatry and obstetrics health, it is crucial to understand better the relationship between maternal glucose levels during pregnancy (specifically, the severity of hyperglycaemia) and its impact on child brain development. Furthermore, the amelioration of significance with adjustment in our study also highlights a need for future studies to elucidate the relationships between factors, such as shared genetics, household income, poorer diet quality, greater obesity, poorer health literacy, lesser educational opportunities and child brain development.

## Conclusion

Metabolic disorders, such as diabetes mellitus before and during pregnancy, are significant public health concerns that pose short and long-term health risks for both the mother and her child. Our study contributes to current knowledge and suggests a possible association between maternal diabetes during pregnancy, externalizing problems in young children aged 4–6 years and ADHD symptoms in children aged 7–10 years. Future research efforts should focus on better understanding the impact of severe metabolic dysregulation before and during pregnancy on early child brain development, to provide insights to improve mother and child health and well-being.

## Supplementary Information

Additional file 1.

## Data Availability

No datasets were generated or analysed during the current study.
