# Supplementary material for: Is maternal diabetes during pregnancy associated with neurodevelopmental, cognitive and behavioural outcomes in children? Insights from individual participant data meta-analysis in ten birth cohorts
Source: BMC Pediatr. 2025 Jan 30;25:76. doi: 10.1186/s12887-024-05365-y (PMC11783732; doi:10.1186/s12887-024-05365-y)
Supplement: Supplementary file 1 — Additional file 1. [file 12887_2024_5365_MOESM1_ESM.docx]

**Supplementary text**

**Content**

[**Supplementary Table 1: Information sources for gestational diabetes** 1](#_Toc184630443)

[**Supplementary text 1: Cohort specific ethic approval and informed consent** 2](#_Toc184630444)

[**Supplementary text 2: Cohort and Author specific funding 5**](#_Toc184630445)

[**Supplementary text 3: Cohort specific Acknowledgement 8**](#_Toc184630446)

# **Supplementary Table 1: Information sources for gestational diabetes**

| **Cohort** | **Source** | **Data type** | **Universal screening** | **Harmonized: Fully or partially** |
| --- | --- | --- | --- | --- |
| **ALSPAC** | Clinical records | Binary | No | Fully |
| **BiB** | OGGT | Binary | Yes | Fully |
| **DNBC** | Clinical Records | Binary | No | Fully |
| **EDEN** | OGGT during study and Clinical records | Binary | Yes | Fully |
| **ELFE** | Clinical Records | Binary | No | Fully |
| **Generation R** | Clinical Records | Binary | No | Fully |
| **MoBa** | Questionnaire | Binary | No | Fully |
| **NINFEA** | Questionnaire | Binary | No | Fully |
| **Raine** | Questionnaire | Binary | No | Fully |

# **Supplementary text 1: Cohort specific ethic approval and informed consent**

This work was supported by The LifeCycle project. This work was supported by EUCAN-connect, a federated FAIR platform enabling large-scale analysis of high-value cohort data connecting Europe and Canada in personalized health. This project received funding from the European Union's Horizon 2020 research and innovation programme under grant agreement No 824989.

**ALSPAC**

Ethical approval for the study was obtained from the ALSPAC Ethics and Law Committee and the Local Research Ethics Committees. Informed consent for the use of data collected via questionnaires and clinics was obtained from participants following the recommendations of the ALSPAC Ethics and Law Committee at the time.

**BiB**

Ethics approval has been obtained for the main platform study and all of the individual substudies from the Bradford Research Ethics Committee. All participants gave written informed consent **DNBC**

The DNBC complies with the Declaration of Helsinki and was approved by the Danish National Committee on Biomedical Research Ethics. Informed consent was obtained from participants upon enrolment.

**EDEN**

The study received approval from the ethics committee (CCPPRB) of Kremlin Bicêtre on 12 December 2002 and from CNIL (Commission Nationale Informatique et Liberté), the French data privacy institution. Women gave written informed consent for themselves and their child. Fathers gave written informed consent for themselves.

**ELFE**

Ethical approvals for data collection in maternity units and for each data collection wave during follow-up were obtained from the national advisory committee on information processing in health research (CCTIRS: Comité Consultatif sur le Traitement de l’Information en matière de Recherche dans le domaine de la Santé), the national data protection authority (CNIL: Comission Nationale Informatique et Liberté) and, in case of invasive data collection such as biological sampling, the committee for protection of persons engaged in research (CPP: Comité de Protection des Personnes). The ELFE study was also approved by the national committee for statistical information (CNIS: Conseil National de l’Information Statistique). Informed consent was signed by the parents or the mother alone, with the father being informed of his right to deny consent for participation.

**Generation R**

The general design, all research aims and the specific measurements in the Generation R Study have been approved by the Medical Ethical Committee of the Erasmus Medical Center, Rotterdam. New measurements will only be embedded in the study after approval of the Medical Ethical Committee. Participants are asked for their written informed consent for the four consecutive phases of the study (prenatally, birth to 4 years, 4–12 years, and from 12 years onwards). At the start of each phase, mothers and their partners receive written and oral information about the study. Even with consent of the parents, when the child is not willing to participate actively, no measurements are performed. From the age of 12 years, children are asked for written informed consent. The current study is based on current data released from the study.

**INMA**

The INMA project was approved by the ethics committee in each area. All participants provided written informed consent before enrolment to the study

**MoBa**

The establishment and data collection in MoBa was previously based on a license from the Norwegian Data protection agency and approval from The Regional Committee for Medical Research Ethics, and it is now based on regulations related to the Norwegian Health Registry Act. MoBa is conducted according to the guidelines laid down in the declaration of Helsinki, and written informed consent was obtained from all participants. A detailed protocol of the study including the consent can be found elsewhere (http://www.fhi.no/morogbarn). The current study is based on version 12 of the quality-assured data files released for research. The establishment of MoBa and initial data collection were based on a license from the Norwegian Data Protection Agency and approval from The Regional Committees for Medical and Health Research Ethics. The MoBa cohort is based on regulations of the Norwegian Health Registry Act. The current study was approved by The Regional Committees for Medical and Health Research Ethics (2018/427). All participants provided written informed consent before enrolment to the study

**NINFEA**

The Ethical Committee of the San Giovanni Battista Hospital and CTO/CRF/Maria Adelaide Hospital of Turin approved the NINFEA study (approval N. 0048362, and subsequent amendments). Informed consent was obtained from all the participants.

**Raine**

Ethics approval was obtained from the Human Ethics Committees at King Edward Memorial Hospital, Princess Margaret Hospital, The University of Western Australia and Curtin University. All participants and guardians provided written consent.

# Supplementary text 2: Cohort and Author specific funding

- **Cohort specific funding**

This work was supported by The LifeCycle project. This project received funding from the European Union's Horizon 2020 research and innovation programme (LIFECYCLE, grant agreement No 733206, 2016).

**Born in Bradford** receives funding from a joint grant from the UK Medical Research Council (MRC) and UK Economic and Social Science Research Council (ESRC) [MR/N024391/1]; the British Heart Foundation [CS/16/4/32482]; a Welcome Infrastructure Grant [WT101597MA]; The National Institute for Health Research under its Applied Research Collaboration for Yorkshire and Humber [NIHR200166].The views expressed are those of the author(s), and not necessarily those of the NHS, the NIHR or the Department of Health and Social Care).

**INMA-Sabadell.** This study was funded by grants from Instituto de Salud Carlos III (Red INMA G03/176; CB06/02/0041; PI041436; PI081151 incl. FEDER funds; PI12/01890 incl. FEDER funds; CP13/00054 incl. FEDER funds; PI15/00118 in cl. FEDER funds; CP16/00128 incl. FEDER funds; PI16/00118 incl. FEDER funds; PI16/00261 incl. FEDER funds; PI17/01340 incl. FEDER funds; PI18/00547 incl. FEDER funds; CPII18/00018), CIBERESP, Generalitat de Catalunya-CIRIT 1999SGR 00241, Generalitat de Catalunya-AGAUR (2009 SGR 501, 2014 SGR 822), Fundació La marató de TV3 (090430), Spanish Ministry of Economy and Competitiveness (SAF2012-32991 incl. FEDER funds), Agence Nationale de Securite Sanitaire de l’Alimentation de l’Environnement et du Travail (1262C0010; EST-2016 RF-21), EU Commission (261357, 308333, 603794 and 634453). We acknowledge support from the grant CEX2018-000806-S funded by MCIN/AEI/ 10.13039/501100011033, and support from the Generalitat de Catalunya through the CERCA Program. The contribution of JRH is supported, in part, by the Research Council of Norway through its Centres of Excellence funding scheme, project number 262700. The Danish National Birth Cohort was established with a significant grant from the Danish National Research Foundation. Additional support was obtained from the Danish Regional Committees, the Pharmacy Foundation, the Egmont Foundation, the March of Dimes Birth Defects Foundation, the Health Foundation and other minor grants.

The **DNBC** Biobank has been supported by the Novo Nordisk Foundation and the Lundbeck Foundation. Follow-up of mothers and children have been supported by the Danish Medical Research Council (SSVF 0646, 271-08-0839/06-066023, O602-01042B, 0602-02738B), the Lundbeck Foundation (195/04, R100-A9193), The Innovation Fund Denmark 0603-00294B (09-067124), the Nordea Foundation (02-2013-2014), Aarhus Ideas (AU R9-A959-13-S804), University of Copenhagen Strategic Grant (IFSV 2012), and the Danish Council for Independent Research (DFF – 4183-00594 and DFF - 4183-00152). Here it is the information of funding in relation to INMA-Gipuzkoa, for the period of study included in the study.

**INMA-Gipuskoa.** This study was funded by grants from Instituto de Salud Carlos III (FIS-PI06/0867, FIS PI09/00090, FIS-PI13/02187, FIS-PI18/01142, FIS-PI18/01237 incl FEDER funds) CIBERESP, Department of Health of the Basque Government (2005111093, 2009111069, 2013111089, 2015111065 and 2018111086), the Provincial Government of Gipuzkoa (DFG06/002, DFG08/001, DFG15/221 and DFG 89/17) and annual agreements with the municipalities of the study area (Zumarraga, Urretxu, Legazpi, Azkoitia y Azpeitia y Beasain). Via the RECONAI platform.

**ELFE cohort** received a government grant managed by the National Research Agency under the "Investissements d'avenir" programme (ANR-11-EQPX-0038 and ANR‑19‑COHO-0001) ». JMDS is funded by Generalitat Valenciana - Regional Ministry of Education, Research, Culture and Sport under the Talented Researcher Support Programme - Plan GenT (CIDEGENT/2019/064).

**EDEN** study was supported by Foundation for Medical Research (FRM), National Agency for Research (ANR), National Institute for Research in Public health (IRESP: TGIR cohorte santé 2008 programme), French Ministry of Health (DGS), French Ministry of Research, INSERM Bone and Joint Diseases National Research (PRO-A) and Human Nutrition National Research Programs, Paris-Sud University, Nestlé, French National Institute for Population Health Surveillance (InVS), French National Institute for Health Education (INPES), the European Union FP7 programmes (FP7/2007–2013, HELIX, ESCAPE, ENRIECO, Medall projects), Diabetes National Research Program (through a collaboration with the French Association of Diabetic Patients (AFD)), French Agency for Environmental Health Safety (now ANSES), Mutuelle Générale de l'Education Nationale complementary health insurance (MGEN), French national agency for food security, French-speaking association for the study of diabetes and metabolism (ALFEDIAM).

**Raine** The Western Australian Pregnancy Cohort (Raine Study) has been funded by program and project grants from the Australian National Health and Medical Research Council, the Commonwealth Scientific and Industrial Research Organisation, Healthway, the Lions Eye Institute in Western Australia and NHMRC EU funding grant GNT114285. The University of Western Australia (UWA), Curtin University, the Raine Medical Research Foundation, the Telethon Kids Institute, the Women’s and Infant’s Research Foundation (KEMH), Murdoch University, The University of Notre Dame Australia and Edith Cowan University provide funding for the Core Management of the Raine Study. REF is a recipient of a National Health and Medical Research Council Early Career Fellowship.

- **Author specific funding**

**Serena Defina** received funding from the European Union’s Horizon 2020 research and innovation programme (grant reference: 848158, EarlyCause). Wenlun Yuan received postdoctoral fellowship from Fondation de France. **Wenlun Yuan**, Team funding (**Barbara Heude, Chloé Vainqueur, Marie aline Charles)** from LONGITOOLS, a project funded by the European Union's Horizon 2020 research and innovation program [grant number 874739**].  Jennifer Ruth Harris** was supported, in part, by the Research Council of Norway through its Centres of Excellence funding scheme, project number **262700**. **Hanan El Marroun** supported by the Stichting Volksbond Rotterdam (HM) and the  the Netherlands Organization for Health Research and Development [Aspasia grant No.015.016.056] (HM). The study sponsors had no role in the study design, collection, analysis and interpretation of data, writing of the report, or in the decision to submit the paper for publication.

# Supplementary text 3: Cohort specific Acknowledgement

The authors acknowledge that **Born in Bradford** is only possible because of the enthusiasm and commitment of the children and parents in Born in Bradford. We are grateful to all participants, health professionals and researchers who have made Born in Bradford happen. We thank the families who have participated in and contributed data to the Norwegian Mother, Father, and Child Cohort Study (**MoBa**).The general design of **the Generation R Study** is made possible by financial support from Erasmus MC, Erasmus University Rotterdam, the Netherlands Organization for Health Research and Development, the Netherlands Organization for Scientific Research, the Ministry of Health, Welfare and Sport, and the Ministry of Youth and Families. The authors sincerely acknowledge the contributions of the participating children, parents, general practitioners, hospitals, midwives, and pharmacies in Rotterdam.

The authors would like to thank the participants, the first Principal Investigator of **DNBC** Prof. Jørn Olsen, the scientific managerial team, and DNBC secretariat for being, establishing, developing and consolidating the Danish National Birth Cohort. **INMA** The authors would particularly like to thank all the participants for their generous collaboration. The authors are grateful to Mireia Garcia, Maria Victoria Estraña, Maria Victoria Iturriaga, Cristina Capo and Josep LLuch for their assistance in contacting the families and administering the questionnaires.
